# Introducing the Multidimensional Toolkit for the Assessment of Play in Schools (M-TAPS): a reliability study

**DOI:** 10.12688/f1000research.160920.1

**Published:** 2025-05-06

**Authors:** Helen Dodd, Rachel Nesbit, Lily FitzGibbon

**Affiliations:** 1 Public Health and Sport Sciences, University of Exeter, Exeter, England, UK; 2 School of Psychology and Clinical Language Sciences, University of Reading, Reading, England, UK; 3 Division of Psychology, University of Stirling, Stirling, Scotland, UK

**Keywords:** play; playtime; recess; measure; observation; child

## Abstract

**Background:**

Despite increasing interest in changing and improving play opportunities in schools, there is lack of openly available methods for evaluating play quantitatively. Existing measures often focus on physical activity during play activities or prioritise the mapping of locations within which play occurs rather than evaluating play itself.

**Methods:**

This paper introduces the Multidimensional Toolkit for the Assessment of Play in Schools (M-TAPS) and provides the results of an initial study examining the utility and reliability of the toolkit. The M-TAPS includes observations of individual children and scan observations of predefined areas of the playground; children’s activities, adventure/risk level and affect are coded. In addition, the M-TAPS includes child self-report questionnaires about emotions during playtime and playtime activities.

**Results:**

The reliability study provided evidence of good reliability between coders and for children’s self-report of their emotions during playtime. There was some indication of validity between child self-report and coder observation.

**Conclusions:**

The paper suggests that the M-TAPS may be useful for research focused on improving children’s play in schools where a quantitative measure is sought. The M-TAPS provides a flexible tool for supporting researchers with the results highlighting how the M-TAPS can provide insights into schools playtimes and how observation can be combined with children’s self-report. There is room for further development and refinement of the toolkit.

## 1. Introduction

Play is intrinsic to childhood and is protected as a fundamental right under the 1989 United Nations Convention on the Rights of the Child (Article 31; UNCRC, 1989). Play offers diverse opportunities for children to express themselves and supports healthy cognitive, social and physical development ( Andersen et al., 2023; Dodd et al., 2023; Herrington & Brussoni, 2015; Nijhof et al., 2018; Singer et al., 2006). There is mounting evidence that, at least in western societies, children’s opportunities for play are diminishing, particularly independent outdoor play ( Clements, 2004; Dodd et al., 2021; Tremblay et al., 2015) and time for play in schools ( Baines & Blatchford, 2019; Henley et al., 2010).

Perhaps in response to declining opportunities for play, a burgeoning area of research focuses on the benefits and importance of play to children’s health, wellbeing, and holistic development ( Jackson et al., 2021; Zhao & Gibson, 2022). Aligned with this, school-based play programmes that aim to increase the diversity and quality of children’s play during school playtimes, are increasingly being delivered and evaluated ( Houser et al., 2019; Johnstone et al., 2018; Lee et al., 2020). School playtimes offer an important context for the promotion and study of play. They offer a play opportunity for all children who are attending school and can therefore reduce inequalities in access to play that may exist outside of school. They offer a unique context where children have dedicated time for play with a wide range of other children. In addition, school playtimes are positively associated with classroom behaviour as well as academic attainment ( Jarrett et al., 1998; Massey, Ku, et al., 2018a; Pellegrini & Bjorklund, 1997; Pellegrini & Bohn, 2005). Given that schools are increasingly expected to support children’s physical activity and mental health, improved play during playtimes may help them to address this need, at least in part.

An example of a play-based programme that has been developed for schools is the Lunchtime Enjoyment And Play (LEAP; Hyndman et al., 2014) intervention which introduces moveable and recycled materials (often referred to as loose parts) to school playgrounds with the aim of increasing physical activity. A similar approach was taken in The Sydney Playground Project ( Bundy et al., 2017), one of the first academic research programmes dedicated to improving play in schools. In the U.K., the Outdoor Play and Learning (OPAL) programme adopts a whole school approach to improving playtime, including the introduction of loose parts, opening up access to space and staff training around play planning and risk ( Lester et al., 2011).

As research develops into the efficacy of school-based play programmes, it is increasingly important to have methods available to support the systematic measurement of play. Play research has a rich tradition of creative, child-centred qualitative, ethnographic and anthropological methods to capture children’s experiences of playtime ( FitzGibbon et al., 2024). These approaches provide unique and valuable insights that cannot, and should not, be replaced by quantitative methods. Nevertheless, for rigorous, empirical evaluation of programmes and comparisons across programmes, it is useful to complement these approaches with quantitative methods.

The quantitative assessment of play is complex and different approaches all have strengths and weaknesses. For example, children’s perspectives on their own play are arguably the most important, and these can be captured in a quantitative way using self-report questionnaires. Nevertheless, children’s perspectives can be strongly influenced by external cues and their most recent experiences, affecting reliability over time. In contrast, observation of children’s play by independent observers can offer an objective perspective but observations can only capture what the observers can see, meaning that children’s internal experiences can be neglected or misinterpreted. Observations also take place over a limited period so what is observed may not always be representative of a typical playtime.

A recent review examined existing measures of play in schools and found that the majority of studies use idiosyncratic measures, often designed for a specific study and only used once ( FitzGibbon et al., 2024). The review highlighted that these measures are rarely available for other researchers to use, and examination of psychometric properties is scarce. There are a few notable exceptions to this such as the Great Recess Framework-Observational Tool ( Massey, Ku, et al., 2018a; Massey, Stellino, et al., 2018b), System for Observing Play and Leisure Activities in Youth (SOPLAY; McKenzie et al., 2000) and the Observation of Playground Play ( Massey, Ku, et al., 2018a). These are robust instruments that have been carefully developed but they are limited in that they either focus on physical activity levels/types of sport, features of the play environment rather than play per se or are designed to measure at the group level, rather than the individual level. This latter point is important for research that aims to map play onto individual difference variables such as age or mental health. It is also vital within an evaluation because it allows researchers to examine how the intervention affects specific subgroups of children, such as those with special education needs. While it has not yet been evaluated for use on school playgrounds, the Tool for Observing Play Outdoors (TOPO; Loebach & Cox, 2020, 2022) has recently been developed as a systematic evaluation protocol for observing children’s play behaviours in outdoor spaces. This protocol has some advantages over those above, particularly the ability to use the tool to assess play at both the individual and group level. However, it is very labour-intensive and has been designed with early-years environments and relatively small groups of children in mind.

In this paper we introduce the Multidimensional Toolkit for the Assessment of Play in Schools (M-TAPS) which is inspired by and complements these existing methods. It includes two observation components and a child self-report component which capture types of play as well as level and type of risk, and children’s affect. The M-TAPS has been designed to be feasible for use in large-scale evaluation; whilst observation of play necessarily requires significant researcher time, some instruments are not practical for use within evaluations because they are extremely labour intensive or require video recordings to be made of school playgrounds, which raises ethical issues. The various components of the toolkit can be used flexibly according to research questions and study aims.

In addition to introducing the measure, in this paper we also present an initial study evaluating the reliability of each component of the M-TAPS, which includes an assessment of whether the level of adventurousness in children’s play and their affect during their play can reliably be coded. Research into the importance of adventurous play (also termed risky play) has been increasing in recent years ( Dodd & Lester, 2021; Sandseter et al., 2023). Adventurous play refers to child-led play involving subjective feelings of excitement, thrill and fear ( Dodd & Lester, 2021; Sandseter, 2009). Despite this increased interest in adventurous play, measures of play in schools rarely include evaluation of this type of play and it remains unclear whether adventurous play can reliably be observed or whether it can only be assessed by asking children themselves.

Similarly, it is unclear whether observers who are not familiar with the children they are observing can reliably code children’s emotions via observation. This is important because play and emotional experience are richly intertwined; for example, Sutton-Smith (2002) argued that one function of play is to help players achieve “emotional joy” ( Sutton-Smith, 2002, p. 19). Play has been described as an ‘emotional toolbox—a safe space in which many emotions can be experienced without consequences or worrying about whether the emotion is acceptable’ ( PALS; 2020). Thus, children’s emotional experience of playtime is an important element of their play experience and ideally an assessment of play should capture this emotional experience.

## 2. Methods

### 2.1 Materials: Multidimensional Toolkit for the Assessment of Play in Schools (M-TAPS)

The M-TAPS was created by the authors and is available at: https://osf.io/qjf8b/. It aims to provide insight into children’s play by combining across child and observer perspectives. Given this, the M-TAPS includes:

1. Scan observations of playtimes, where the number of children engaging (or not) in certain types of play, including adventurous play, and the number of children displaying positive and negative affect are counted. The scan observations provide an overview of play across the playground.
2. Focal observations of specific children whose play, adventure level and affect are observed more closely and coded. Focal observations provide insight into individual children’s play and can be linked to individual difference factors or used to capture changes in children’s of play over time.
3. A child-report questionnaire pack that includes a questionnaire about their emotions during playtime and a questionnaire about their activities during playtime.

These three components have been designed to be complementary; they can be combined but each could be used in isolation, depending on specific research questions. Each component will now be described in turn.

**2.1.1 Scan observations**

For the scan observations, playgrounds are divided into defined areas which allow the researchers to be stationary and view the entire area without any significant obstructions. The number of areas required will vary by school but each area should be scanned at least twice. We recommend that observers visit the school to select the areas and pilot data collection with those areas ahead of starting the scan observations. A complete round of scan observations is then conducted for each area before moving to the next area. During each round of scan observations, observers count the number of children doing and not doing each of the following types of play (see extended data for full definitions for each code – available at: https://osf.io/qjf8b/):

1. Playing (yes/no)
2. Sport (yes/no)
3. Fixed equipment/markings (yes/no)
4. Active/chase (yes/no)
5. Nature/landscape (yes/no)
6. Rough and Tumble (yes/no)
7. Small world/Toys (yes/no)
8. Sport/play equipment (yes/no)
9. Loose parts/recycled materials (yes/no)
10. Antisocial (yes/no)

These categories of play were initially developed based on those included in the OPP ( Massey, Ku, et al., 2018a) and aligned with the activities on the Activities During Playtime measure (see 2.1.3). However, following discussion with adults familiar with UK playgrounds and piloting, some edits were made: we added ‘markings’ to the equipment category, added ‘landscape’ to the nature category and added two additional categories of ‘sport/play equipment’, ‘loose parts/recycled materials’. In addition, we removed ‘traditional playground games’ because during piloting it became clear that defining this was challenging given different childhood experiences of coders.

Alongside these play categories we also explored the extent to which adventure level and affect could be coded during playground scans. This was motivated by the growing interest in adventurous play and a desire to capture children’s emotional experience if possible. Thus, the following were also counted during scan observations (see extended data for full definitions for each code):

11. High adventure (yes/no)
12. At least moderate adventure (yes/no)
13. Affect (positive/negative/unclear)

Observers scan from left to right imagining a straight line moving across the space. Each child is counted as the imaginary line reaches them and, for each of the play categories above, is classified as doing (yes) or not doing (no), each activity. For example, an initial scan is completed from left to right with all children categorised as playing/not playing. Then a new scan begins left to right counting the number of children playing sport and so on until all categories have been coded. For all categories children are counted as doing the activity/adventure level (yes) or not doing it (no) with the exception of affect which is coded as positive, negative or unclear. Once scans have been completed for all categories, that round is complete and observers move to the next area and repeat.

**2.1.2 Focal observations**

Specific children are selected for focal observations. These children can be selected at random or according to demographic or other characteristics depending on the purpose of the study. Observers first locate a focal child on the playground and then begin the observation. The observer observes the child for 10 seconds and then records:

1. Type of play (using the play categories listed for the scan observations above with ‘not playing’ instead of playing and with ‘social play’ and ‘other play’ added as options). Social play was added during piloting of the focal observations to capture children chatting or hanging out with friends. This was not included in the scans for this study because they were completed first but can be included in scan observations within future research if desired.
2. Level of adventure (High/Moderate/Low). If level of adventure is coded as moderate or high then the type of risk is also subsequently coded (Height, speed, impact, tools, rough and tumble, alone, vicarious, dangerous elements). These risk categories are based on previous research ( Kleppe et al., 2017; Sandseter, 2009).
3. Affect (positive/negative/unclear). The child’s affect is coded as positive or negative, only if it is clearly one or the other (e.g. a child giving a broad smile or a child crying), otherwise unclear was given.

Observers are given 15 seconds to record this information and then the next 10 second observation period begins. For this study, observers completed 15 × 10 second observations per focal child before finding the next focal child and beginning their observation.

**2.1.3 Child-report questionnaire pack**

The child report questionnaire pack includes two questionnaires.

***Emotions during playtime.*** An adapted version of the Positive and Negative Affect Scale for Children (PANAS-C; Laurent et al., 1999) was created to capture the emotions that children experience during school playtimes. The PANAS-C asks children to respond using a 5-point Likert scale (from ‘Not much or not at all’ to ‘A lot’) the extent to which they have experienced each of 30 emotions over the previous 2 weeks. To create the M-TAPS *Emotions During Playtime* measure we adapted the PANAS-C. The emotions and response scale are identical to the original measure but children are asked to respond to each item thinking about how they have felt during playtime over the past week. The PANAS-C was selected as the basis for the measure because of the broad range of positive and negative emotions included and because it is a well-established measure with strong psychometric properties ( Laurent et al., 1999). Note that although the PANAS-C originally included 30 items, following Laurent and colleagues, only 27 items contribute to the positive and negative affect scales with *alert, fearless* and *daring* excluded from the scale scoring. We included all 30 items to support future research which could explore emotional profiles of playtimes and also to consider whether individual items might give useful insights. The PANAS-C also exists as a 10-item measure ( Ebesutani et al., 2012) so a 10-item version of the Emotions During Playtime measure could be used if only an approximate measure of overall positive and negative affect during playtime is needed. Given that children’s report of their emotions may be affected by their state emotion, the Emotions During Playtime questionnaire is designed to be completed on two occasions at least 2 days apart, with responses averaged across the two completions.

***Activities during playtime.*** A second questionnaire was developed to capture what activities children say they do during playtimes. Children were asked to think about school break and lunchtimes over the past two weeks and for each of eight activities state how much they do the activity. Activities were: playing sports; playing on fixed play equipment or markings; playing with loose objects; playing with nature; playing chase games or running around; play fighting or wrestling; fighting, arguing or trying to break things; not playing (examples were provided). Children responded on a three-point scale: ‘Not at all’, ‘A little’, ‘A lot’. These categories of play were developed based on those included in the Observation of Playground Play (OPP; Massey, Ku, et al., 2018a). At the end of the questionnaire, children were asked to write any other types of play and their favourite activity.

### 2.2. Methods: Reliability study

**2.2.1 Participants**

To evaluate the reliability of each part of the M-TAPS, we recruited participants from a primary school located in Bristol, UK. The school was located in a relatively low-income urban area where residents are predominantly white; the school population and study participants reflect this demographic. Data were collected in March 2022.

For child self-report, all children from one class in each of years 3, 4 and 5 respectively (aged 7-10 years) were invited to participate. We chose to include only children aged 7 years and above given the reading and cognitive capacity required to respond to the questionnaires. In total 62 children started the questionnaire pack.

Scan observations were conducted across the entire playground meaning that all children from years 1 to 6 (aged 5 to 11 years) were included in these observations. In the UK, children attend primary school from age 4 to age 11 (or a combination of infant and junior school). Age 4-5 is termed Reception. These children follow a different curriculum to the older children and their playgrounds are often separate from the rest of the school. Given this, we chose to focus on children from Year 1 upwards but we have no reason to believe that the M-TAPS could not be used with younger children.

Focal observations were conducted with 44 children (22 male, 22 female) selected at random. We did not collect any identifying information about these children but, because children at this school have playtimes staggered by age, we know that a range of ages were included. Of the focal children, 13 were in Year 1 (aged 5 or 6 years), 17 in Years 2, 3 or 4 (aged 6 to 9 years), and 14 in Years 5 or 6 (aged 9 to 11 years).

For an inter-rater ICC of .8 (against a null of.5), a sample of 37 gives 99% power to detect a significant association between two raters and 95% power to detect a significant association across two points, therefore the sample size was adequate to address the primary aims related to assessing reliability ( Walter et al., 1998).

Following consultation with the appropriate ethical review board, we chose to use an opt-out consent procedure. The school provided proxy informed consent for all participants and parents were given detailed information about the study with options to withdraw their child. No parent chose to remove their child from the study. The benefit of this approach is that a representative sample can be obtained and typical playtimes, with all children on the playground, could be observed. A limitation of this approach is that, to minimise any risks associated with opt-out consent, all data were collected anonymously. This means that no personal or demographic information was collected about individual participants.

**2.2.2 Procedure**

The methods and study procedures were approved by the University of Reading Research Ethics Committee (Ref: 2021-163-RM) on 15th November 2021. The study adhered to the Declaration of Helsinki principles. Parents were provided with written information about the research and asked to inform the school or research team if they did not want their child to complete questionnaires and/or be on the playground during the observations. A range of response options were given including emailing, phoning or texting the school or research team. Children were given information about the study via a short, pre-recorded presentation and asked to provide assent before completing questionnaires. They were also given opportunity to ask their teacher and/or the researchers questions. Children were given paper copies of the questionnaire packs to complete during class time under the supervision of their class teacher and learning support assistant. The pre-recorded presentation told children that the questionnaires should be completed alone and that they should not discuss the answers with other children. Teachers were also given written information from the research team asking them to create test conditions for the completion of the questionnaires. Time 1 data collection consisted of two sessions (completed an average of 2.30 days apart (range 0-12 days)). During the first, all questionnaires were completed once. In the second session just the Emotions During Playtime measure was completed. At Time 2 (which began an average of 5.51 days after Time 1 (range 0-10 days), the same procedure was followed (the two sessions at Time 2 were completed an average of 5.16 days apart (range 2-12 days)).

For the scan observations, the research team viewed the playground and divided it into five defined areas during a pre-visit. Data collection was then piloted to ensure that these areas were appropriate and to confirm standing positions for the coders. This process also gave children time to get used to the observers being on the playground and reduced the likelihood that the presence of the observers would affect children’s play. In total 15 area scans were completed by both observers with each area scanned at least twice (area 1 = 6 scans; area 2 = 3 scans; area 3 = 2 scans; area 4 = 2 scans; area 5 = 2 scans). Note that the Year 1 children were only allowed to use area 1 for their playtime so this area was scanned more frequently. Observers synced the start time of each scan to ensure they were observing the same play, for the purpose of assessing reliability.

For the focal observations, the coders selected a child to observe at random and coordinated the start of the observation to ensure they were both observing the child at the same time. Where specific children need to be observed, we have piloted using coloured wrist-worn sweatbands to identify those children and this approach has worked well. Children wear the sweatbands on the outside of any sleeves or coats so that they are visible and each child is assigned a colour during the coding period.

After coordinating the start of scan and focal observations, coders did not consult with one another about what codes to allocate to ensure that the reliability assessments were accurate and to avoid observers biasing one another. Scan and focal observations were supported by an app developed for the purpose of this research. The app was programmed in Microsoft PowerApps and can store data locally on a tablet/phone that is later transferred to secure databases within the Microsoft SharePoint environment. The materials required to create a usable instance of the app, including instruction videos and data templates, are available here: https://osf.io/qjf8b/.

**2.2.3 Missing data**

The questionnaire booklet was started by 62 children and the questionnaires were completed by the following numbers of children: Emotions During Playtime Positive Affect scale (T1 = 53; T2 = 53; T3 = 49; T4 =43); Emotions During Playtime Negative Affect scale (T1 = 54; T2 = 53; T3 = 48; T4 = 43); Activities During Playtime questionnaire (responses varied from 53 responses to 56 responses for each activity at T1 and from 47 responses to 50 responses at T2). For the focal observations, of 660 possible individual observation data points (15 per child x 44 children) a minority were missing, primarily due to technical errors or interruptions during coding: 11 for activity, 17 for adventure, and 20 for affect. Analyses are conducted with available data.

## 3. Results

All analyses were conducted in R Studio version 2023.12.1 running R version 4.3.3 ( R Core Team, 2024). For the three components of the M-TAPS our primary aim was to examine reliability. For scan and focal observations, we focused on inter-rater reliability and used intra-class correlation coefficient (ICC) calculated the irr package ( Gamer et al., 2019) in R ( R Team, 2021) to examine consistency between raters on continuous variables and Cohen’s Kappa using the irr package in R for categorical data. For ICCs we examined absolute agreement using two-way random effects models with reliability estimated for a single rating. For questionnaires, we examined test-retest reliability using Concordance Correlation Coefficient (CCC) as measure of reliability for continuous data and Cohen’s kappa for categorical data. CCC is preferable to Pearson correlations because it captures both precision and accuracy. The CCCs were calculated using the DescTools ( Signorell, 2023) package in R. For questionnaires where items are summed to create scales we also examined internal consistency via Cronbach’s alpha using the ltm package ( Rizopoulos, 2006) in R. The analysis code, output and raw data are openly available via this link: https://osf.io/qjf8b/.

### 3.1 Scan observations

**3.1.1 Activity**

A total of 15 scans were conducted by each rater. During the scans each visible child in the area being coded was coded as engaging (or not) in each activity in turn. Table 1 shows each activity and the minimum, maximum and mean percentage of participants coded as engaging in each activity. The minimum and maximum values relate to single scans (i.e. 0% indicates that during at least one scan no child was observed engaging in that activity), with the mean values showing the mean percentage across all of the scans. The majority of children observed were coded as engaged in play (mean = 86.2%), with the most popular activities being playing with sports or play equipment followed by active play or chase. ICCs ranged from 0.73 to 1.

**Table 1.** Minimum, mean, and maximum percentage of scan observations coded for each activity by rater, with ICC.

	Rater 1			Rater 2			ICC
	Min	Mean	Max	Min	Mean	Max	
*Activity*							
Playing	57.1%	86.2%	100.0%	71.4%	89.3%	100.0%	0.73
Sports/play equipment	0.0%	29.6%	81.8%	0.0%	30.8%	81.8%	0.99
Active/chase	0.0%	17.1%	92.3%	0.0%	16.8%	91.7%	0.98
Sport	0.0%	12.4%	100.0%	0.0%	13.2%	100.0%	1.00
Small world/toys	0.0%	9.5%	28.6%	0.0%	11.0%	36.0%	0.96
Loose parts/recycled materials	0.0%	6.8%	50.0%	0.0%	7.9%	57.1%	0.98
Rough and tumble	0.0%	2.4%	12.9%	0.0%	2.2%	13.3%	0.94
Fixed equipment/markings	0.0%	2.3%	9.1%	0.0%	1.6%	6.2%	0.83
Nature/landscape	0.0%	1.2%	13.3%	0.0%	1.0%	15.4%	0.95
Antisocial	0.0%	0.0%	0.0%	0.0%	0.0%	0.0%	
*Adventure level*							
Moderate	0.0%	24.9%	83.3%	0.0%	23.1%	66.7%	0.80
High	0.0%	0.0%	0.0%	0.0%	0.0%	0.0%	
*Affect*							
Positive	0.0%	10.0%	26.7%	0.0%	9.7%	22.9%	0.76
Negative	0.0%	1.6%	11.8%	0.0%	0.6%	5.6%	0.73
Unclear	73.3%	88.5%	100.0%	77.1%	89.7%	100.0%	0.78

**3.1.2 Adventure**

During the scan observations, each child’s adventure level was also coded. As shown in Table 1 only around one-quarter of children were observed to be playing with at least moderate adventure during scan observations. No children were coded by either observer to be playing with high adventure during scans. ICC for proportion of children playing with at least moderate adventure was 0.80 (see Table 1).

**3.1.3 Affect**

During the scan observation, each child’s affect was also coded. For the majority of children affect was coded as unclear with only a very small minority of children expressing any clear negative affect. The ICC values ranged from 0.73 to 0.76 (see Table 1).

### 3.2 Focal observations

Focal observations were made by each rater independently. Each rater coded the activity, adventure level and affect they observed during each observation period. For information, the proportions of total observations coded for each activity, adventure level and affect coded across all participants are shown in Table 2, split by rater. The minimum and maximum values relate to single participants (i.e. 0% indicates that at least one child did not engage in that activity at all during their focal observation, and 100% indicates that at least one child engaged in that activity throughout their focal observation), with the mean values showing the mean percentage across all of participants. Table 3 also shows the proportion of adventurous play codes that were assigned to each type of risk. Note that risk categories were only available when raters coded adventure as moderate or high, so these proportions are from a total of 45 observations for Rater 1 and 48 observations for Rater 2.

**Table 2.** Percentage of focal observations coded under each activity, adventure level and affect label, by rater with ICC.

	Rater 1			Rater 2			ICC
	Min	Mean	Max	Min	Mean	Max	
*Activity*							
Sports/play equipment	0.0%	31.4%	100.0%	0.0%	32.3%	100.0%	1.00
Social play	0.0%	23.5%	100.0%	0.0%	22.8%	93.3%	0.97
Active/chase	0.0%	7.8%	46.7%	0.0%	7.3%	46.7%	0.98
Loose parts/recycled materials	0.0%	5.5%	100.0%	0.0%	5.3%	93.3%	1.00
Nature/landscape	0.0%	4.9%	66.7%	0.0%	4.4%	60.0%	0.98
Small world/toys	0.0%	4.4%	60.0%	0.0%	4.4%	60.0%	1.00
Fixed equipment/markings	0.0%	2.5%	26.7%	0.0%	2.7%	26.7%	0.98
Sport	0.0%	1.5%	66.7%	0.0%	1.5%	66.7%	1.00
Rough and tumble	0.0%	1.2%	20.0%	0.0%	1.4%	20.0%	0.90
Other play	0.0%	0.5%	7.1%	0.0%	0.5%	7.1%	1.00
Antisocial	0.0%	0.8%	26.7%	0.0%	0.8%	26.7%	1.00
Not playing	0.0%	16.1%	73.3%	0.0%	16.7%	73.3%	0.97
*Adventure level*							
Low	46.7%	92.6%	100.0%	40.0%	92.7%	100.0%	0.95
Moderate	0.0%	7.2%	53.3%	0.0%	7.1%	60.0%	0.96
High	0.0%	0.2%	10.0%	0.0%	0.2%	10.0%	1.00
*Affect*							
Negative	0.0%	1.7%	26.7%	0.0%	1.4%	26.7%	0.96
Positive	0.0%	14.8%	100.0%	0.0%	17.0%	100.0%	0.96
Unclear	0.0%	83.5%	100.0%	0.0%	81.7%	100.0%	0.96

**Table 3.** Percentage of focal observations coded under each risk category, by rater. Note that risk category was only coded when adventure level was moderate or high.

	Rater 1	Rater 2
*Risk*		
Speed	46.7%	45.8%
Impact	26.7%	22.9%
Height	24.4%	27.1%
Rough and tumble	2.2%	2.1%
Other	0.0%	2.1%

Raters gave the same activity code on 96% of observations, Kappa = 0.95. For adventure level, raters agreed on 98.4% of observations, Kappa = 0.89. For affect, raters agreed on 96.4% of observations, Kappa = 0.88. For risk categories, only Height, Impact, Speed, Routh and Tumble and other were observed; raters agreed on 97.6% of the 41 observations where both coders categorised play as moderate or high adventure, Kappa = 0.96. The ICCs for all focal observation categories were excellent, ranging from 0.9-1 (see Table 2).

### 3.3 Questionnaires

Section 2.2.3 shows the number of children who completed each questionnaire.

**3.3.1. Emotions during playtime**

The Emotions During Playtime scales were completed twice at Time 1 and twice at Time 2 as detailed in 2.1.3 and 2.2.2. Table 4 displays the mean and standard deviation for the positive affect score and negative affect score for the Emotions During Playtime measure, at each completion point. Scores based on the 27-item version and the 10-item version (to align with the different versions of the PANAS-C) are included for comparison purposes. The values show that positive affect was slightly higher than negative affect across all completion points and that scores were slightly lower for both positive and negative affect when only 10 items were used.

**Table 4.** Mean and standard deviation for positive and negative affect scores based on the Emotions During Playtime measure as calculated using 27-items and 10-items.

Scale and completion point	27-item version		10-item version	
	Mean	(SD)	Mean	(SD)
Positive Affect (T1)	2.98	(1.09)	2.97	(0.9)
Positive Affect (T2)	3.20	(1.21)	3.11	(1.04)
Positive Affect (T3)	3.22	(1.14)	2.95	(0.94)
Positive Affect (T4)	3.18	(1.15)	2.95	(1.04)
Negative Affect (T1)	2.17	(0.97)	2.09	(0.79)
Negative Affect (T2)	2.23	(1.01)	2.15	(0.88)
Negative Affect (T3)	2.14	(1.09)	2.06	(0.94)
Negative Affect (T4)	2.01	(1.05)	1.88	(0.92)

Our a priori intention was to average across the two completions at each timepoint to give an indication of emotions experienced during playtime. The test-retest reliability when this approach was used was good (CCC = 0.87 for positive affect and CCC = 0.81 for negative affect with 27 items; CCC = 0.82 for positive affect and CCC = 0.81 for negative affect with 10 items). To explore whether this approach was required or whether the consistency between a single completion of the Emotions During Playtime measure at Time 1 and Time 2 would be adequate the CCCs for the first Emotions During Playtime measure completed at T1 and the first completed at T2 were evaluated. These were weaker (CCC = 0.7 for positive affect and CCC = 0.55 for negative affect for 27 items; CCC = 0.72 and CCC = 0.56 for 10 items). This suggests that completing the Emotions During Playtime measure on two occasions gives a more reliable estimate of both positive and negative affect.

Cronbach’s alpha was calculated for the positive affect and negative affect scales independently at each completion point, with complete data only and with all data. For positive affect, alpha values ranged from 0.80 – 0.90 and, for negative affect, alpha values ranged from 0.87-0.92 (for the 10-item scale, values ranged from 0.73 – 0.83 for positive affect and 0.73 – 0.85 for negative affect).

As described previously, we included all 30 items to support future research which could explore emotional profiles of playtimes and also to consider whether individual items might give useful insights. We therefore also evaluated test-retest reliability for each item (see Table 6). The majority of the items showed reasonable consistency over time and sufficient variation that they could be used in isolation in future research if needed. Notable exceptions were ‘happy’, ‘disgusted’, ‘miserable’, ‘strong’ and ‘gloomy’ which had poor consistency.

**3.3.2 Activities during playtime**

Table 5 shows the proportion of children who responded that they played each activity ‘Not at all’, ‘A little’ or ‘A lot’ during playtime. At both timepoints the most commonly reported activities were sport and chase/tag. Test-retest reliability regarding specific activities was relatively poor; Kappas ranged from 0.13 – 0.41 for the consistency of responses. We also examined whether reliability would be stronger if we collapsed ‘A little’ and ‘A lot’ responses to give a binary variable but reliability was only slightly improved with Kappas from 0.19 to 0.54 (see Table 5).

**Table 5.** Proportion of children selecting each response on the Activities During Playtime questionnaire. Kappa (3) shows the Kappa value when all three categories were included. Kappa (2) shows the Kappa value when ‘A little’ and ‘A lot’ were combined.

		Time 1	Time 2	Kappa (3)	Kappa (2)
Sport	Not at all	33.9%	16.0%	0.40	0.54
	A little	30.4%	32.0%		
	A lot	35.7%	52.0%		
Fixed Equipment	Not at all	39.3%	42.9%	0.13	0.19
	A little	44.6%	34.7%		
	A lot	16.1%	22.4%		
Loose parts	Not at all	47.3%	58.3%	0.35	0.37
	A little	27.3%	29.2%		
	A lot	25.5%	12.5%		
Nature	Not at all	42.6%	46.9%	0.36	0.33
	A little	42.6%	28.6%		
	A lot	14.8%	24.5%		
Chase, tag	Not at all	12.5%	16.0%	0.26	0.19
	A little	41.1%	36.0%		
	A lot	46.4%	48.0%		
Play fighting	Not at all	54.5%	60.0%	0.26	0.43
	A little	38.2%	30.0%		
	A lot	7.3%	10.0%		
Fighting, arguing, breaking things	Not at all	64.3%	61.7%	0.26	0.28
	A little	21.4%	29.8%		
	A lot	14.3%	8.5%		
Not playing	Not at all	40.0%	47.9%	0.41	0.47
	A little	41.8%	29.2%		
	A lot	18.2%	22.9%		

**Table 6.** Concordance Correlation Coefficient (reliability) by item for the Emotions During Playtime questionnaire. Scores are averaged across two completions at T1 and two completions at T2, lower and upper 95% confidence intervals also shown.

Item	N	Mean	St. Dev.	Min	Max	CCC	CCC lwr.ci	CCC upr.ci
Interested	52	2.577	1.526	1	5	0.68	0.44	0.83
Sad	55	2.291	1.474	1	5	0.62	0.37	0.79
Frightened	56	1.714	1.057	1	4	0.70	0.48	0.83
Alert	52	2.327	1.167	1	5	0.59	0.30	0.77
Excited	53	3.019	1.538	1	5	0.71	0.51	0.84
Ashamed	52	1.769	1.198	1	5	0.74	0.56	0.86
Upset	53	2.245	1.329	1	5	0.66	0.41	0.81
Happy	54	3.481	1.563	1	5	-0.25	-0.43	-0.06
Strong	53	3.094	1.656	1	5	-0.06	-0.37	0.26
Nervous	54	2.185	1.361	1	5	0.63	0.37	0.80
Guilty	54	1.741	1.031	1	5	0.64	0.41	0.79
Energetic	53	3.358	1.618	1	5	0.85	0.72	0.93
Scared	54	2.148	1.485	1	5	0.65	0.39	0.81
Calm	53	2.811	1.481	1	5	0.66	0.41	0.81
Miserable	54	2.13	1.428	1	5	0.35	0.04	0.60
Jittery	51	2.098	1.33	1	5	0.55	0.26	0.75
Cheerful	54	2.889	1.562	1	5	0.80	0.63	0.89
Active	54	3.204	1.583	1	5	0.73	0.52	0.86
Proud	55	2.836	1.549	1	5	0.58	0.30	0.77
Afraid	55	1.927	1.274	1	5	0.72	0.50	0.85
Joyful	52	3	1.547	1	5	0.81	0.63	0.91
Lonely	53	2.17	1.411	1	5	0.69	0.42	0.84
Mad	55	2.4	1.382	1	5	0.75	0.53	0.87
Fearless	54	2.5	1.437	1	5	0.67	0.41	0.83
Disgusted	55	1.982	1.408	1	5	0.40	0.06	0.66
Delighted	53	2.623	1.457	1	5	0.64	0.38	0.81
Blue	53	2.226	1.436	1	5	0.82	0.65	0.91
Daring	52	2.327	1.581	1	5	0.65	0.39	0.82
Gloomy	52	2.327	1.618	1	5	0.32	-0.04	0.61
Lively	53	2.66	1.652	1	5	0.52	0.21	0.74

## 4. Discussion

Our primary aim was to introduce the M-TAPS and describe it in such a way that it could be utilised in future research when combined the materials we have made openly available. In addition, we aimed to examine the reliability of each of the components of the M-TAPS. Specifically, we examined inter-rater reliability for the scan and focal observations and test-retest reliability as well as internal consistency, where appropriate, for the child self-report measures. We also explored whether adventure and affect could be coded effectively. Each component is now discussed in turn.

For the scan and focal observations, reliability estimates fell in the good to excellent range for most of the activities as well as for adventure levels. The only exception was the reliability of affect categorisation during scan observations which was in the moderate range. Overall, very little adventure and negative affect were coded which raises the question of whether these codes are worthwhile retaining in future studies. The M-TAPS is designed to be flexible and users can decide whether they wish to code adventure and affect. Our own reflections are that the low levels of affect and adventure are due to different underlying issues. The coders reported that affect was very difficult to evaluate based only on appearance. This aligns with research showing that facial expressions do not always align with internal emotional state ( Barrett et al., 2019). The low rate of negative and positive affect coded was likely, therefore, due to uncertainty regarding emotion; it may therefore not be worthwhile attempting to code affect in future studies, particularly if children are completing the Emotions During Playtime measure, where they were able to provide relatively reliable self-report of emotions. In contrast, the low levels of moderate and high adventure during the observed playtimes seem to be an accurate representation of the adventure levels present. If a school made their playtimes more adventurous then we anticipate that the observation components of the M-TAPS would be able to capture these higher levels of adventure. Future research will need to explore this further to be certain though. Given the very low amount of high adventure coded, the current results do not allow us to conclude whether high adventure can reliably be coded but the reliability estimate for the ‘at least moderate adventure’ code allows us to be optimistic that adventure level can be captured objectively to some extent.

The test-retest reliability and internal consistency for the Emotions During Playtime measure, which is based on the PANAS-C ( Ebesutani et al., 2012; Laurent et al., 1999) were good when the measure was completed twice at time 1 and twice at time 2. We planned to ask children to complete the measure twice and to average responses at both time points because we anticipated that children’s feelings about playtime on any particular day may affect responses. To examine whether this approach was necessary, we also evaluated the test-retest reliability of a single completion at Time 1 and Time 2. This fell in the moderate range and was notably lower than for the average of two completions at both timepoint. We therefore recommend that the measure is completed twice where practical. Given that the PANAS-C was originally designed with 30 items, which were subsequently reduced to 27 items and then to 10 items for the short-form, we examined the reliability and internal consistent for both the 27-item version and the 10-item version. The results showed that the 10-item version gave slightly lower scores on average and had slightly lower internal consistency, but the two versions were relatively consistent with one another overall. The 10-item version may therefore be preferred in future research.

In contrast to the Emotions During Playtime measure, the Activities During Playtime Questionnaire showed poor test-retest reliability at the individual child level. There are a number of possible explanations for this. First, it is possible that children are heavily influenced by what they did during their most recent playtime and therefore aren’t able to give a reliable response about what they typically do over a two-week period. Second, children’s play activities may not be stable over time, so children may be providing valid responses but the low test-retest reliability may indicate that their play activities change over time. At the group level, the results from the Activities During Playtime questionnaire were somewhat consistent over time; chase/tag and sport were the most commonly played ‘a lot’ at both time points and the least frequently played ‘a lot’ at both time points were play fighting and fighting, arguing, breaking things. In contrast, the rankings of nature, loose parts and fixed equipment across the two timepoints were less consistent. This may therefore indicate that children are quite consistent in their playing of chase/tag or sport during playtimes but less consistent in their play with loose parts, fixed equipment and nature, perhaps moving between these activities over time. It is informative that the most common play activities observed during both scan and focal observations were also ‘using sport or play equipment’ and ‘active play or chase’ (with social play also being frequently observed during focal observations). Very little nature play, rough and tumble play or loose parts play was observed. This provides some initial validity across the components of the M-TAPS and supports the interpretation that children are consistently engaging in these types of play.

Although our aim was to evaluate reliability of the M-TAPS components, the results demonstrate how the measure can provide insights into children’s play during school playtimes. In addition to highlighting the popularity of play with sports/play equipment and active play/chase, the results show that whilst a high proportion of children were engaged in play during the observations around 11-14% of children were not playing. From discussion with the coders there were various reasons for this. For example, a child may be walking between activities or may be looking around to decide what to do next. In addition, some children were observed to be disengaged or withdrawn from play. A further insight is that high levels of adventure were almost never observed and moderate levels of adventure were observed only between 10% and 25% of the time across the different types of observation. This is consistent with research showing that schools in the UK are hesitant about allowing adventurous play during playtime ( Nesbit et al., 2021).

The study was designed as an initial evaluation of the reliability of the M-TAPS. There are therefore some limitations to consider. First, it may be useful to add in the perspective of school staff, especially those who supervise lunch and breaktimes; these members of school staff may be able to provide insight into trends in play that may not be captured during discreet periods of observation. Second, the reliability study was conducted within one school so should be considered preliminary; further evaluation across a range of schools would be ideal to ensure that the results can be generalised. This would also allow the factor structure of the Emotions During Playtime measure to be evaluated, which could not be conducted in this study due to power limitations. Furthermore, we have not evaluated test-retest reliability of the observation components. A final limitation to consider is that we focus on reliability rather than validity. Certainly, there is some evidence of validity due to the consistency across the M-TAPS components in terms of the most common and most rare activities, but the study was not designed as a rigorous evaluation of validity.

## 5. Conclusion

The M-TAPS provides a flexible tool for supporting researchers to capture children’s play in schools in a quantitative way. The results highlight how the M-TAPS can provide insights into schools playtimes and the need for multiple perspectives; integrating observation with children’s self-report. An initial psychometric evaluation provides support for the various components, although child report of playtime activities may not be reliable at the individual child level. Suggestions of various options for researchers are included such as the decision of whether to evaluate affect during observations, and ideas and directions for further evaluation of the instrument are described. We anticipate that the M-TAPS will complement the rich methods available for qualitative approaches children’s play and existing measures of play in schools.

## Ethics and consent

The methods and study procedures were approved by the University of Reading Research Ethics Committee (Ref: 2021-163-RM) on 15 ^th^ November 2021. The study adhered to the Declaration of Helsinki principles. Parents were provided with written study information sheets via their child’s school and were asked to inform the school or the study team if they did not want their child to participate. This written opt-out consent procedure was approved by the research ethics panel because all of the data collected for the study was anonymous and because the school gave permission for the data to be collected during school hours. No personal data was collected and it was not possible for the research team to link any data to a child’s name or other identifiable information.

## CRediT statement

Conceptualization, H.D., R.N., L.F.; methodology, H.D., R.N., L.F.; software, n/a; validation, H.D., L.F.; formal analysis, H.D., L.F.; investigation, H.D., R.N., L.F.; resources, H.D., R.N., L.F.; data curation, H.D., L.F., writing—original draft preparation, H.D.; writing—review and editing, H.D., L.F., R.N.; visualization, L.F.; supervision, H.D.; project administration, H.D.; funding acquisition, H.D. All authors have read and agreed to the published version of the manuscript.

## Data Availability

OSF: Multidimensional Toolkit for the Assessment of Play in Schools (M-TAPS). https://osf.io/qjf8b/ ( Dodd et al., 2025). This project contains the following underlying data:
- Scan data_raw.csv (raw data from scan observations) - focal obs data_reliability_wide.csv (raw data from focal observations) - Raw child data practice school_questionnaires.csv (raw data from child questionnaires) Data is available under the terms of the https://creativecommons.org/licenses/by/4.0/deed.en (CC-By Attribution 4.0 International) Code for replicating the analyses in R Studio available from: https://osf.io/qjf8b/ All materials used for the M-TAPS, including the app coding and the questionnaires, alongside the coding protocol, are available alongside the data at: https://osf.io/qjf8b/.
