# Supplementary material for: Introducing the Multidimensional Toolkit for the Assessment of Play in Schools (M-TAPS): a reliability study
Source: F1000Res. 2025 Oct 20;14:476. Originally published 2025 May 6. [Version 2] doi: 10.12688/f1000research.160920.2 (PMC12596566; doi:10.12688/f1000research.160920.2)
Supplement: Peer review history

# Reviewer report: Reviewer response for version 2

**DOI:** 10.5256/f1000research.189788.r425681

**Published:** 2025-12-11

**Authors:** Oliver Traynor

**Affiliations:** 1 University of Glasgow, Glasgow, Scotland, UK

This paper addresses an important gap in play research by developing a standardised scalable and openly available assessment toolkit. This paper is likely to be of interest to researchers wishing to quantitively evaluate play interventions and children’s play behaviours in school settings and can complement qualitative approaches. I have structured the following suggestions in line with the paper’s headings.

**Introduction**

 The authors provide a good background to the study and cite relevant literature.

**Methods**

 The authors acknowledge that the reliability testing of the tool is conducted within one school, in a demographically narrow setting. This could be emphasised more strongly in the discussion with reference to how context such as school-level policies, playground structure, and cultural norms may affect reliability and category relevance.

 Some categories overlap conceptually such as “sport”, “sport/play equipment”, “active/chase.” Clarification would help the reader understand how coders differentiate between these.

**Results**

 Given that high adventurous play was not observed and negative affect was rarely observed, the authors could discuss plans for future validation in settings that offer more adventurous play.

 A sizeable proportion of children were coded as “not playing” with reasons including transition between activities and social withdrawal. I question whether transition between activities should be classified as “not playing.” I think this requires more nuance and recommend that future work should distinguish transitions from genuine disengagement – I think TOPO categorises these behaviours well.

**Conclusion**

 The paper presents a valuable tool and strong initial reliability. The openness of the materials and methodological transparency are key strengths and will contribute to advancing the field of assessing children’s play behaviours. I hope the authors find my comments useful.

Is the rationale for developing the new method (or application) clearly explained?

Yes

Is the description of the method technically sound?

Yes

Are the conclusions about the method and its performance adequately supported by the findings presented in the article?

Yes

If any results are presented, are all the source data underlying the results available to ensure full reproducibility?

Yes

Are sufficient details provided to allow replication of the method development and its use by others?

Yes

Reviewer Expertise:

Children's play behaviours, the influence of the natural environment on health behaviours, developing and evaluating complex interventions.

I confirm that I have read this submission and believe that I have an appropriate level of expertise to confirm that it is of an acceptable scientific standard.

# Reviewer report: Reviewer response for version 2

**DOI:** 10.5256/f1000research.189788.r425684

**Published:** 2025-11-08

**Authors:** Sylvia Fernandez Rao

**Affiliations:** 1 ICMR National Institute of Nutrition, Hyderabad, India

This manuscript covers the development and initial reliability testing of the Multidimensional Toolkit for the Assessment of Play in Schools (M-TAPS), a promising instrument combining scan observations, focal observations, and child self-report measures.

 The paper makes a valuable contribution to the growing literature on school-based play assessment. However, before indexing, some parts require clarification and strengthening regarding generalisability, validity evidence, statistical reporting, and treatment of missing data.

 The reliability testing was undertaken in a single UK primary school serving a somewhat homogeneous, low-income, primarily white community. While this is acknowledged, the constraint might be explained more plainly.

 Please comment on how the school context (staggered playtimes, playground size/layout, staff supervision, and risk policies) might have influenced both the observed prevalence of adventurous play and inter-rater reliability. It would be helpful to note that psychometrics may not generalise to schools with various sociodemographic circumstances, playground systems, or cultural attitudes toward risk.

 The focus on reliability is good for an initial review, but at times the manuscript indicates that M-TAPS is already validated. It would strengthen the manuscript to more clearly divide what has been established (reliability) from what has to be tested (validity).

 The manuscript says that studies were undertaken “with available data,” but details are lacking. Missing data may affect reliability estimations if not random also describe how missing data were handled.

 The Activities During Playtime questionnaire demonstrates low test–retest reliability at the individual level authors highlighted plausible causes (state effects, variability), but it would help to translate this into practical guidance for future researchers.

 Briefly explain coder training, calibration, and blinding techniques to help readers determine feasibility.

 Correct minor typos (“Routh and Tumble” for “Rough and Tumble”) and verify all references and table captions are complete.

 Overall, this is a good, well-implemented work that provides a valuable methodological contribution. With additional clarity on generalisability, validity, and missing data, the manuscript will be appropriate for indexing.

 I recommend a minor revision focused on clarity and refining assertions.

Is the rationale for developing the new method (or application) clearly explained?

Yes

Is the description of the method technically sound?

Yes

Are the conclusions about the method and its performance adequately supported by the findings presented in the article?

Yes

If any results are presented, are all the source data underlying the results available to ensure full reproducibility?

Yes

Are sufficient details provided to allow replication of the method development and its use by others?

Partly

Reviewer Expertise:

Early child development

I confirm that I have read this submission and believe that I have an appropriate level of expertise to confirm that it is of an acceptable scientific standard.

# Reviewer report: Reviewer response for version 1

**DOI:** 10.5256/f1000research.176881.r384603

**Published:** 2025-06-03

**Authors:** Maeghan James

**Affiliations:** 1 Children's Hospital of Eastern Ontario, Ottawa, Ontario, Canada

Thank you for the opportunity to review this manuscript titled: “Introducing the Multidimensional Toolkit for the Assessment of Play in Schools (M-TAPS): a reliability study”. In this paper, the authors introduce a novel tool for assessing play in schools and present reliability statistics of the tool. The authors concluded that some components of the M-TAPS tool were reliable and provide important considerations for future observational work using this tool. Overall, the paper was very well written – clear, concise and informative. The authors raise an important point regarding the need for more universal observational tools that can suit the needs of a variety of research projects. I believe the topic is of importance and will be a valuable contribution to the literature. Below I outline some questions/comments I had about the work that I believe, once addressed, would strengthen the paper.  

1. One of the activity categories is labeled as “playing,” but many of the listed activities would arguably fall under the general concept of play. It is unclear how “playing” is being specifically defined in contrast to other categories such as “active/chase.” Clarifying the definitions and providing examples would help distinguish between these overlapping categories. This is primarily why I indicated “partly” to whether sufficient details were provided to allow replication of the method development and its use by others. This information may have been somewhere on the OSF link but it wasn’t working for me so I could not determine if there was enough information on the codes themselves to allow for replication. I recommend including the full list of codes and their definitions either as a table within the manuscript or as a supplementary file.
2. Given the subjective nature of observational data, I appreciate the inclusion of affect and adventurous play categories. However, it is essential that the tool and/or codebook explicitly challenge normative assumptions about what play looks like. For example, what  “positive affect” or “adventurous play” looks like. This is particularly relevant for children and youth with diverse abilities and backgrounds. This could be addressed through specific training or guidance notes in the codebook.
3. The results section suggests that most activities were coded under the general “playing” category. It remains unclear whether coders were allowed to select multiple categories (e.g., “playing” and “sports/play equipment”). If coders could only choose one, this may lead to a loss of context and detail regarding the type of play observed.
4. The low ICC value for the “playing” category is notable. Could this be due to coders needing to choose between “playing” and a more specific type of activity, even when both might be appropriate? This suggests potential ambiguity in the coding decision process. A possible alternative approach could involve first determining whether a behavior constitutes “play” (yes/no), followed by a second level of coding to classify the type of play. This hierarchical strategy may improve reliability and clarity.
5. While ICC values for affect (when coded as positive or negative) were strong, the majority of affective observations were coded as “unclear.” This suggests that identifying emotional expression via observation alone may be inherently limited. I appreciate the authors’ reflection on the challenges of coding affect. Rather than removing the affect category, it may be more productive to consider how visible indicators of emotion (e.g., facial expressions, body language) could be systematically coded to aid decision-making.
6. The strong agreement among coders is encouraging, but it raises the question of whether these classifications are valid. Although validating affect codes was not the purpose of this study, it would be valuable to know if there are plans to evaluate this in future research. This may also warrant further discussion beyond the limitations section to acknowledge the inherent biases associated with observational research and to clarify that, in its current form, the tool cannot yet be considered validated which has important implications for practice.
7. For the Activities During Playtime Questionnaire, it would be helpful to describe what measures were taken to ensure that children consistently understood each activity type (e.g., nature play vs. free play). It is possible that variability in children’s interpretations affected their responses? Were examples or visual aids provided to support consistent understanding at both time points?
8. When discussing the reliability findings for the PANAS-C, it may strengthen the discussion to include a brief comparison with reliability estimates reported in previous literature, if available.

Is the rationale for developing the new method (or application) clearly explained?

Yes

Is the description of the method technically sound?

Yes

Are the conclusions about the method and its performance adequately supported by the findings presented in the article?

Yes

If any results are presented, are all the source data underlying the results available to ensure full reproducibility?

Yes

Are sufficient details provided to allow replication of the method development and its use by others?

Partly

Reviewer Expertise:

Observational research on children’s active and outdoor play, with a focus on child development, childhood disability, and the use of systematic observation protocols in playground settings.

I confirm that I have read this submission and believe that I have an appropriate level of expertise to confirm that it is of an acceptable scientific standard, however I have significant reservations, as outlined above.

# Response

**Published:** 2025-10-15

**Authors:** Helen Dodd

**Affiliations:** Public Health & Sports Science, University of Exeter, Exeter, England, UK

Thank you for your thought-provoking review of our manuscript. In the revised version, we have clarified the reporting of the observational protocols and included a table to address several of the issues you have raised. We also include now benchmark our reliability findings against relevant previous findings, as suggested. We respond to each of your comments in turn below.

 1. One of the activity categories is labeled as “playing,” but many of the listed activities would arguably fall under the general concept of play. It is unclear how “playing” is being specifically defined in contrast to other categories such as “active/chase.” Clarifying the definitions and providing examples would help distinguish between these overlapping categories. This is primarily why I indicated “partly” to whether sufficient details were provided to allow replication of the method development and its use by others. This information may have been somewhere on the OSF link but it wasn’t working for me so I could not determine if there was enough information on the codes themselves to allow for replication. I recommend including the full list of codes and their definitions either as a table within the manuscript or as a supplementary file.

***Response: **We appreciate having the lack of clarity around the categories for the scan observations drawn to our attention. Scans were done one category at a time, so were not designed to be mutually exclusive of one another. For example, the first scan “Playing” simply sought to determine the proportion of children on the playground who were considered to be playing, rather than engaged in some non-play activity such as eating, observing, or walking between areas of the playground. This first scan encompasses all kinds of play. Later scans identified whether specific types of play were occurring. For focal observations, the “playing” category was replaced with “not playing” and an “other play” category was also included so that play that did not fit into the other categories could be distinguished from non-play behaviour. We now make that clearer and, following your suggestion, we now include a table of definitions for each category (Table 1) that shows the response options for scan and focal observations side by side for easy comparison.*

 2. Given the subjective nature of observational data, I appreciate the inclusion of affect and adventurous play categories. However, it is essential that the tool and/or codebook explicitly challenge normative assumptions about what play looks like. For example, what  “positive affect” or “adventurous play” looks like. This is particularly relevant for children and youth with diverse abilities and backgrounds. This could be addressed through specific training or guidance notes in the codebook.

***Response: **This is a very important point that we agree has not yet been well addressed in our research, or indeed elsewhere. We now include this as a discussion point with a call for future research to evaluate the coding scheme with children with a diverse range of abilities and backgrounds.*

 3. The results section suggests that most activities were coded under the general “playing” category. It remains unclear whether coders were allowed to select multiple categories (e.g., “playing” and “sports/play equipment”). If coders could only choose one, this may lead to a loss of context and detail regarding the type of play observed.

***Response: **Again, thank you for drawing our attention to the lack of clarity relating to the selection of the categories. For scan observations, each category was scanned separately, so categories were independent. In each scan, the coder simply thought about the category they were scanning for, say “playing” and considered whether each child was playing or not in that moment. One would expect that the proportion of children observed to be “playing” would be higher than for each of the sub-categories of play, and indeed for these sub-categories combined. This is not necessarily the case since the scans occur consecutively, and children may change activity between scans. Indeed, in our data, the proportion of children “playing” in the scan observations was similar to the proportion of children in the other play categories combined, suggesting that these categories did indeed describe most of the kinds of play children engaged in. For focal observations, there was no “playing” category, but if the child’s activity did not fit one of the play categories, they could choose whether they were engaged in “other play” and write some notes about that play afterwards, or “not playing”. We have clarified this in the manuscript and in Table 1.*

 4. The low ICC value for the “playing” category is notable. Could this be due to coders needing to choose between “playing” and a more specific type of activity, even when both might be appropriate? This suggests potential ambiguity in the coding decision process. A possible alternative approach could involve first determining whether a behavior constitutes “play” (yes/no), followed by a second level of coding to classify the type of play. This hierarchical strategy may improve reliability and clarity.

***Response: **As outlined in the previous comment, the coders did not need to chose between “playing” and specific types of play/activity, so the lower ICC is not due to this. Instead, this lower ICC may be due to the definition of ‘play’ being more ambiguous than the specific types of play, and/or because the quick pass nature of scan observations does not give sufficient time for the presence/absence of play to be established. Consistent with this explanation, reliability of the category “not playing” in the focal observations was near perfect, suggesting that with more time observing a child, coders can more consistently evaluate whether a child is playing or not. We suggest that future researchers are cautious about using broad and subjective categories such as “playing” for scan observations where more time might be required to understand the context of a child’s behaviour (see also discussion of coding affect during scan observations). It is nonetheless useful to determine an approximate estimate of the number of children engaged in play activity because low estimates might be a key indicator of problems with a particular playground environment.*

 5. While ICC values for affect (when coded as positive or negative) were strong, the majority of affective observations were coded as “unclear.” This suggests that identifying emotional expression via observation alone may be inherently limited. I appreciate the authors’ reflection on the challenges of coding affect. Rather than removing the affect category, it may be more productive to consider how visible indicators of emotion (e.g., facial expressions, body language) could be systematically coded to aid decision-making.

***Response: **It is true that there was a high incidence of the ‘unclear’ category being used by the coders. We now reflect upon this in more detail in the discussion, with reference to the prevenance of positive and negative emotions reported in the self-reported Emotions During Playtime measure.*

 6. The strong agreement among coders is encouraging, but it raises the question of whether these classifications are valid. Although validating affect codes was not the purpose of this study, it would be valuable to know if there are plans to evaluate this in future research. This may also warrant further discussion beyond the limitations section to acknowledge the inherent biases associated with observational research and to clarify that, in its current form, the tool cannot yet be considered validated which has important implications for practice.

***Response: **Note that we had already stated “*there is some evidence of validity due to the consistency across the M-TAPS components in terms of the most common and most rare activities, but the study was not designed as a rigorous evaluation of validity”. *In light of the reviewer's previous comment though, we have further considered validity in the final paragraph of the discussion.*

 7. For the Activities During Playtime Questionnaire, it would be helpful to describe what measures were taken to ensure that children consistently understood each activity type (e.g., nature play vs. free play). It is possible that variability in children’s interpretations affected their responses? Were examples or visual aids provided to support consistent understanding at both time points?

***Response: **We have realised that the questionnaire packs were not available in the supplementary materials at the time of the original submission. The full questionnaire pack is now available in the supplementary materials. Given the limited explanation of the categories provided to children, we now suggest that poorer reliability of some categories may indicate a lack of understanding in the discussion and suggest that visual aids may improve the validity of children’s responses.* *We have added further detail about the protocol for the self-report questionnaire packs.*

 8. When discussing the reliability findings for the PANAS-C, it may strengthen the discussion to include a brief comparison with reliability estimates reported in previous literature, if available.

***Response: **We now include comparison of the Emotions During Playtime internal consistency and test-retest reliability with previous PANAS-C findings.*

# Reviewer report: Reviewer response for version 1

**DOI:** 10.5256/f1000research.176881.r384599

**Published:** 2025-05-29

**Authors:** Lisa Barnett

**Affiliations:** 1 Deakin University, Melbourne, VIC, Australia

This is a well written article, and it focuses on the need for new measurement in the field - which I agree with. The introduction provides a good background to the study and cites relevant literature.

 In terms of methods, I am familiar with the SOFIT and SOPLAY tools and have undertaken modifications of these instruments myself for other purposes. One modification in the literature is the CAST instrument tested in the Australian environment and developed for children. CAST2 is a modified version still using momentary time sampling that was used to measure children’s physical activity levels in a school playground environment. CAST2 uses continuous 75 second scans of the break period, which they argued might be more reliable than intermittent scans. Can you comment on the duration used in your study for the scans and why this length of time was chosen?

 When I first read the initial part of the methods, I was dubious about being able to scan for affect and this was what was found in the results. The thing about these scanning tools is it must be very easy to see the behaviour for whole groups of children at once. That is why it works to detect physical activity behaviour such as standing or walking. But to assess affect requires a look at the children's face and a judgement and I don't think this is feasible when trying to document what large groups of children are doing in a quick observation. Antisocial also falls into this camp as how can this be quickly decided? The other categories of observation all make sense to me. I like the idea of focal observations of particular children to gain a more in-depth insight. I think affect would be more able/feasible to be coded for a focal child rather than for the broad scan. 

 Can you please provide some psychometric information for the PANAS-C tool? Your adaptation rests on this tool but you have not provided evidence for it.

 For the activities during playtime tool you may have also wished to consult this paper as a starting point as the team used a pictorial format for the children: Parrish, A. M., Iverson, D., Russell, K., & Yeatman, H. (2010). The Development of a Unique Physical Activity Self-Report for Young Children: Challenges and Lessons Learned. Research in Sports Medicine, 18(1), 71–83. https://doi.org/10.1080/15438620903423874 Also, similar to above, in this section no psychometric information for the OPP tool is provided.

 For participants – can you give an idea how many children were on the playground at the same time? How many were in the school population?

 How exactly were the children selected at random for the focal point?

 Lucky you could use an opt out ethics!

 Can you comment in the discussion on whether a mean of five days apart for the measures is a good measure of reliability?

 How many observers/raters were there?

 Results

 The reliability scores are good

 The discussion is a good overview, and I agree on the findings with regard to the affect observations.

 To me it makes sense that emotions during playtime scale might be less reliable as emotions are less reliable than behaviours such as physical activity and we would assume that thee could change form playtime to playtime.

 Were images used in the Activities during playtime Questionnaire ? would be interesting to see if the use of images or not affect reliability with children.

 I agree with conclusion statement.

Is the rationale for developing the new method (or application) clearly explained?

Yes

Is the description of the method technically sound?

Yes

Are the conclusions about the method and its performance adequately supported by the findings presented in the article?

Yes

If any results are presented, are all the source data underlying the results available to ensure full reproducibility?

Yes

Are sufficient details provided to allow replication of the method development and its use by others?

Yes

Reviewer Expertise:

Motor skills, physical literacy, play, children, instrument development

I confirm that I have read this submission and believe that I have an appropriate level of expertise to confirm that it is of an acceptable scientific standard, however I have significant reservations, as outlined above.

# Response

**Published:** 2025-10-15

**Authors:** Helen Dodd

**Affiliations:** Public Health & Sports Science, University of Exeter, Exeter, England, UK

Thank you for your thorough and thoughtful review of our manuscript. We believe that the clarity of reporting is much improved thanks to your suggested revisions. Below we respond point-by-point to each of your comments and suggestions.

 1. In terms of methods, I am familiar with the SOFIT and SOPLAY tools and have undertaken modifications of these instruments myself for other purposes. One modification in the literature is the CAST instrument tested in the Australian environment and developed for children. CAST2 is a modified version still using momentary time sampling that was used to measure children’s physical activity levels in a school playground environment. CAST2 uses continuous 75 second scans of the break period, which they argued might be more reliable than intermittent scans.  

***Response: **Thank you for bringing this to our attention. We now include the CAST2 in our list of previous measures of playground observation tools.*

 2. Can you comment on the duration used in your study for the scans and why this length of time was chosen? 

***Response: **For pragmatic reasons, the scans are not timed, but rather occur consecutively, beginning the next scan category after the last has finished. The playground is divided so that the scans were completed over an area that was visible to the researcher without them needing to move. The number of children scanned may vary between areas and between scans, so the length of time required for a scan to take place may also vary. Each area of the playground is scanned for each category consecutively. It is recommended that each area is scanned at least twice. We have revised the manuscript to clarify aims and practicalities of the scan observations.*

 3. When I first read the initial part of the methods, I was dubious about being able to scan for affect and this was what was found in the results. The thing about these scanning tools is it must be very easy to see the behaviour for whole groups of children at once. That is why it works to detect physical activity behaviour such as standing or walking. But to assess affect requires a look at the children's face and a judgement and I don't think this is feasible when trying to document what large groups of children are doing in a quick observation. Antisocial also falls into this camp as how can this be quickly decided? The other categories of observation all make sense to me. I like the idea of focal observations of particular children to gain a more in-depth insight. I think affect would be more able/feasible to be coded for a focal child rather than for the broad scan.  

***Response: **We agree that there are practical challenges assessing the affect of children quickly and from a distance (i.e., during scan observations), as reflected in the poorer inter-rater reliability. It is also the case that children’s facial expressions do not always align with their internal emotions, and that children did not themselves report particularly frequent experiences of emotions during playtimes in the self-report measure. This is now discussed in more detail.*

 4. Can you please provide some psychometric information for the PANAS-C tool? Your adaptation rests on this tool but you have not provided evidence for it. 

***Response: **We now include a more detailed description of the psychometric properties of the PANAS-C as well as the link to the original validation study.*

 5. For the activities during playtime tool you may have also wished to consult this paper as a starting point as the team used a pictorial format for the children: Parrish, A. M., Iverson, D., Russell, K., & Yeatman, H. (2010). The Development of a Unique Physical Activity Self-Report for Young Children: Challenges and Lessons Learned. Research in Sports Medicine, 18(1), 71–83. https://doi.org/10.1080/15438620903423874  

***Response: **This is now included in the discussion where we consider improvements for future research.*

*6. *Also, similar to above, in this section no psychometric information for the OPP tool is provided. 

***Response: **ICCs of the OPP observational tool reported in Massey et al. (2018) are now reported. No further psychometric information is available.*

 7. For participants – can you give an idea how many children were on the playground at the same time? How many were in the school population? 

***Response: **More details about the number of children in the school population and across the different parts of the study are now included in the Method.*

 8. How exactly were the children selected at random for the focal point? 

***Response: **The researchers were on the playground and opportunistically selected a child to observe immediately before starting each focal observation. This is not strictly ‘random’ so we have edited the wording.*

*9. *Can you comment in the discussion on whether a mean of five days apart for the measures is a good measure of reliability? 

***Response: **The five-day timescale of the test-retest reliability assessment was fairly short in this study, we have added this to the limitations.*

 10. How many observers/raters were there? 

***Response: **There were two coders who completed all of the coding. They were trained by the first author. This is now stated explicitly.*

 11. Results

 The reliability scores are good 

***Response: **Thank you*

 12. The discussion is a good overview, and I agree on the findings with regard to the affect observations. 

***Response: **Thank you*

 13. To me it makes sense that emotions during playtime scale might be less reliable as emotions are less reliable than behaviours such as physical activity and we would assume that they could change form playtime to playtime. 

***Response: **Child report of emotions was actually more reliable than their reporting of activities. Note that we asked children to complete the emotions measure twice because we expected some fluctuation. Our reflections on this therefore remain largely unchanged in the revised manuscript and are found.   * 

 14. Were images used in the Activities during playtime Questionnaire ? would be interesting to see if the use of images or not affect reliability with children. 

***Response: **The suggestion to include pictorial cues for each activity is now included in the discussion. We also realised that the questionnaire packs were not made available in the Supplementary materials on OSF, these are now available.*

 15. I agree with conclusion statement. 

***Response: **Thank you, this remains unchanged after revision of the manuscript.*
